# Exercise-induced FNDC5/irisin protects nucleus pulposus cells against senescence and apoptosis by activating autophagy

**DOI:** 10.1038/s12276-022-00811-2

**Published:** 2022-07-26

**Authors:** Wenxian Zhou, Yifeng Shi, Hui Wang, Linjie Chen, Caiyu Yu, Xufei Zhang, Lei Yang, Xiaolei Zhang, Aimin Wu

**Affiliations:** 1 Department of Orthopaedics, The Second Affiliated Hospital and Yuying Children’s Hospital of Wenzhou Medical University, 325000 Wenzhou, Zhejiang Province China; 2 Zhejiang Provincial Key Laboratory of Orthopedics, 325000 Wenzhou, Zhejiang Province China; 3 The Second School of Medicine, Wenzhou Medical University, 325000 Wenzhou, Zhejiang Province China; 4 The School of Ophthalmology and Optometry, Wenzhou Medical University, 325000 Wenzhou, Zhejiang Province China; 5 Laboratory Animal Center, Wenzhou Medical University, 325000 Wenzhou, Zhejiang Province China

**Keywords:** Endocrinology, Diseases, Bone

## Abstract

Intervertebral disc degeneration (IVDD) is a major cause of low back pain (LBP), and excessive senescence and apoptosis of nucleus pulposus (NP) cells are major pathological changes in IVDD. Physical exercise could effectively delay the process of intervertebral disc degeneration; however, its mechanism is still largely unknown. Irisin is an exercise-induced myokine released upon cleavage of the membrane-bound precursor protein fibronectin type III domain-containing protein 5 (FNDC5), and its levels increase after physical exercise. Here, we show that after physical exercise, FNDC5/irisin levels increase in the circulation and NP, senescence and apoptosis are reduced, autophagy is activated in NP tissue, and the progression of IVDD is delayed. Conversely, after knocking out FNDC5, the benefits of physical exercise are compromised. Moreover, the overexpression of FNDC5 in NP tissue effectively alleviated the degeneration of the intervertebral disc (IVD) in rats. By showing that FNDC5/irisin is an important mediator of the beneficial effects of physical exercise in the IVDD model, the study proposes FNDC5/irisin as a novel agent capable of activating autophagy and protecting NP from senescence and apoptosis.

## Spinal disease: Exercise-induced protein protects against disc degeneration

Physical exercise in rodents helps to promote expression of a protein that guards against intervertebral disc degeneration (IVDD), a major cause of lower back pain. A team from Wenzhou Medical University in China, led by Xiaolei Zhang and Aimin Wu, showed that mice and rats that participated in regular swimming sessions were less prone to IVDD, an effect mediated by a protein called irisin. Increased levels of this protein (and its precursor form, known as FNDC5) in the cells of the jelly-like tissue found in the core of spinal discs helped to enhance cellular recycling processes. This in turn prevented cell death and deterioration in the spine. Boosting the activity of FNDC5/irisin, either through drugs or exercise, could therefore help alleviate back pain and delay the progression of IVDD in humans.

## Introduction

Low back pain (LBP), one of the most common diseases, is the main cause of disability worldwide and brings a huge economic burden to society, and more than 80% of people have experienced LBP in their lifetime^1–4^. A previous study has shown that 87% of patients with persistent low back pain have intervertebral disc degeneration (IVDD)^5^.

The intervertebral disc (IVD) is the soft tissue located between the vertebrae, consisting of the nucleus pulposus (NP), annulus fibrosus (AF), and cartilage endplates^6^. Gelatinous NP tissue is the main tissue that maintains the structural stability and biomechanical balance of the intervertebral disc, and dysfunction and hypocellularity of NP cells are considered to be hallmarks of IVDD^7,8^. Pathological factors such as inflammatory cytokines and oxidative stress can induce the production of reactive oxygen species (ROS), and the excessive production of ROS leads to abnormal apoptosis and senescence of NP cells^9^. Therefore, intervention in the apoptosis and senescence of NP cells may be an effective strategy for the treatment of IVDD^10,11^.

Physical exercise is beneficial to human health and is related to reducing the risk of mortality^12^. In the reviews by Henchoz et al. and Owen et al., physical exercise was concluded to reduce the pain of patients with LBP and improve their physical functions^13,14^. In terms of cells, physical exercise could effectively promote the cell proliferation of NP and AF, thereby promoting the repair of IVDs^15–17^. Currently, most studies explain the benefits of physical exercise on IVDD in terms of mechanical stimulation^17^, muscle strength^18,19^, and nutritional metabolism^20,21^. However, the molecular mechanism underlying the beneficial effect of physical exercise on IVDD has yet to be elucidated.

Irisin is a myokine released into the circulation during physical exercise and is capable of promoting fat metabolism and thermogenesis, which is hydrolyzed from fibronectin type III domain-containing protein 5 (FNDC5)^22–24^. In previous studies, FNDC5/irisin increases the autophagy level of chondrocytes and inhibits oxidative stress and chondrocyte apoptosis, thereby mitigating the development of osteoarthritis^25,26^. In addition, irisin can inhibit the apoptosis of osteocytes^26,27^, and the vertebral subchondral bone plays a crucial role in the function of the spine^28^. These findings increase the possibility that FNDC5/irisin exerts a cytoprotective effect in degenerative diseases such as IVDD.

In this study, we investigated the changes in FNDC5/irisin in the plasma and NP after physical exercise in mice and rats and verified the hypothesis that FNDC5/irisin may be a key mediator of the beneficial effects of physical exercise on the senescence and apoptosis of NP cells in IVDD models, thereby becoming a potential target for IVDD therapeutic intervention.

## Materials and methods

### Physical exercise

Six-month-old mice and twelve-month-old rats were adapted to swimming for 10 min each day for 2 days to reduce water-induced stress. The duration of exercise was gradually increased until mice and rats swam for 60 min, which was reached, on average, on the 5th day of training. Exercise sessions were performed during the light cycle and consisted of 60 min swimming sessions, 7 days per week for 4 weeks. Mice swam in groups of four in plastic barrels (52 cm depth × 44 cm diameter), and rats swam in groups of two in plastic barrels (87 cm depth × 70 cm diameter). The water temperature was maintained at ~24 °C. Animals were sacrificed via anesthetization 1 h after the last test session^29^.

### X-ray imaging analysis

X-ray examinations were performed on animals in all groups at 0 and 4 weeks. A digital X-ray machine (Kubtec Model XPERT.8; KUB Technologies Inc.) was used to perform X-ray imaging of mice at 50 kV and 160 μA to evaluate the disc height. The disc height index (DHI) was determined using the published method^30^. DHI was expressed as the mean of three measurements from the midline to the central 50% disc width boundary divided by the mean of the heights of two adjacent vertebral bodies. Changes in DHI for punctured discs were expressed as a percentage (%DHI = postpunctured DHI/prepunctured DHI × 100).

### ELISA

Tissue samples were homogenized by ultrasound in 1X PBS and supplemented with protease inhibitor. Blood was obtained by cardiac puncture, collected in low-protein adsorption plastic tubes coated with heparin, and centrifuged. The plasma fractions were either fresh for ELISA or immediately frozen in liquid nitrogen and stored at −80 °C until analysis. Irisin was detected by an irisin ELISA kit (E-EL-M2743c and E-EL-R2625c, Elabscience) according to the manufacturer’s instructions after sample dilution optimization.

### Histology and immunohistochemistry

The tail discs of mice and rats were fixed with 4% paraformaldehyde, decalcified with 0.5 M EDTA, and embedded in paraffin. The sample was cut into 5-μm-thick sections, and the sections were deparaffinized with xylene, hydrated with graded ethanol, and stained with hematoxylin and eosin (HE) and safranin-O (SO). The construction of the IVD and the morphology and cellularity of NP cells were observed by three observers under blinded conditions using a microscope (Leica), and the histological score was evaluated according to the grading scale^30,31^. Cleaved-caspase3, p16INK4a, LC3B, and p-AMPK were detected by immunohistochemical staining of mouse and rat disc sections, as performed using the following antibodies: rabbit polyclonal anti-cleaved-caspase3 (1:400 dilution, #9661, Cell Signaling Technology (CST)); rabbit polyclonal anti-p16INK4a (1:200 dilution, A0262, ABclonal); mouse monoclonal anti-LC3B (1:200 dilution, #83506, CST); and rabbit monoclonal anti-p-AMPK (1:200 dilution, #2535, CST).

### Rat NP cell culture

Rat NP cells were extracted from healthy NP of young Sprague-Dawley rats (150–200 g). NP tissues were isolated by microscopy and digested with 0.25% type II collagenase (Solarbio) at 37 °C for 2 h. Then, the digested tissues were cultured in DMEM/F12 (Gibco) with 10% fetal bovine serum (FBS; Gibco) and antibiotics (1% streptomycin/penicillin) in an incubator at 5% CO_2_ at 37 °C.

### Western blotting

NP cells were lysed with RIPA lysis buffer containing protease and phosphatase inhibitor cocktail (NCM Biotech). The following antibodies were used for western blotting: rabbit monoclonal antibodies: (GAPDH, 1:1000 dilution, #5174, CST; p62, 1:1000 dilution, ab240635, Abcam; FNDC5, 1:1000 dilution, ab174833, Abcam; p-AMPK, 1:1000 dilution, #2535, CST; AMPK, 1:1000 dilution, #5832, CST; p-mTOR, 1:1000 dilution, #5536, CST; mTOR, 1:1000 dilution, #2983, CST); rabbit polyclonal antibodies: (cleaved-caspase3 1:1000 dilution, #9661, CST; p16INK4a 1:1000 dilution, A0262, ABclonal); mouse monoclonal anti-LC3B (1:1000 dilution, #83506; CST).

### qRT–PCR

Total RNA was extracted from NP cells, rat NP, spleen, and muscle by the TRIzol method (Invitrogen). Total RNA was reverse transcribed using a PrimeScript-RT kit (Takara) and a CFX96 real-time PCR system (Bio-Rad Laboratories). The resulting cDNA was amplified by SYBR Premix Ex Taq using the CFX96 real-time PCR system (Bio-Rad Laboratories) and PCR primers summarized in Supplementary Table 1. For the target gene, the transcript levels were normalized with respect to that of GAPDH and evaluated using the 2^−△△Ct^ method.

### Cell viability assay

The Cell Counting Kit-8 (CCK-8; Dojindo Co.) was used to detect the effect of TBHP on the viability of NP cells. According to the manufacturer’s protocol, the NP cells were treated with different concentrations of TBHP (0, 20, 40, 60, 80, and 100 μM) for 24 h. Then, the cells were washed with PBS and incubated with 100 μl of DMEM/F12 medium containing 10% CCK-8 solution for 2 h at 37 °C. The absorbance was measured at 450 nm using an ultraviolet spectrophotometer (Thermo Fisher). All experiments were performed in triplicate.

### Lentiviral transfection

NP cells reaching 40–60% confluence were transfected using LV-FNDC5 lentivirus (NM_001270981; GeneChem) or LV-NC lentivirus at a multiplicity of infection (MOI) of 50. After 12 h of transfection, the culture medium was changed every other day. When confluent, the transfected NP cells were passaged for further experiments.

### Cell treatment

NP cells reaching 70–80% confluence were treated with or without 0, 50, 100, and 200 ng/ml irisin for 24 h and 60 μM TBHP for 24 h; the above-transfected NP cells reaching 70–80% confluence were treated with or without 60 μM TBHP for 24 h. Subsequently, the above cells were used for experimental detection.

### Immunofluorescence

NP cells were plated in a six-well plate. After treatment, NP cells were washed three times with PBS, fixed with 4% paraformaldehyde, and then infiltrated with 0.1% Triton X-100 in PBS. After blocking the cells with 5% bovine serum albumin, they were incubated with mouse monoclonal anti-LC3B (1:1000 dilution, #83506, CST). Finally, the cells were incubated with Alexa Fluor 594-conjugated secondary antibodies (715–585–150; Jackson ImmunoResearch) and stained with the nuclear staining dye DAPI. A Zeiss LSM800 confocal microscope was used to observe the stained cells in five random different fields for each slide.

### Apoptosis assay

Terminal deoxynucleotidyl transferase dUTP nick-end labeling (TUNEL) staining was used to detect the level of DNA damage in NP cells. According to the manufacturer’s protocol, the NP cells were fixed and stained with an in situ cell death detection kit (G3250; Promega Corporation) at 37 °C for 30 min, and the nuclei were stained with DAPI. Twenty-five fields of view were randomly selected from each slide, and TUNEL-positive cells were counted under a Nikon ECLIPSE Ti microscope.

### SA-β-gal staining

The level of senescence was measured by an SA-β-gal staining kit (C0602; Beyotime) according to the manufacturer’s protocol. Aging cells showing high SA-β-gal activity were stained blue. Twenty-five fields of view were randomly selected from each slide, and SA-β-gal-positive cells were counted under a Nikon ECLIPSE Ti microscope.

### Animal models

Adult male Sprague–Dawley rats (1-month-old) were purchased from the Experimental Animal Institute of Wenzhou Medical University. The experimental level rat tail disc (Co7/8) was located by digital palpation on the coccygeal vertebrae and confirmed by counting the vertebrae from the sacral region in a trial radiograph. Needles (27 G) were used to puncture the whole layer of AF from the tail skin. Then, 5 μl of LV-NC or LV-FNDC5 (virus concentration: 10^9^ TU/ml) was injected into the central space of the NP using a 27 G needle. The rats were monitored daily to ensure their health. All animals could free unrestricted weight bearing and activity and were fed food and water regularly.

### Magnetic resonance imaging (MRI)

Four weeks after the operation, the rats were examined by MRI to assess the degree of IVDD. As described in a previous study, all rats were subjected to magnetic resonance imaging using a 3.0 T clinical magnet to assess the signal and structural changes in the sagittal T2-weighted image. The degree of IVDD was evaluated by a blinded orthopedic researcher using the Pfirrmann MRI grading system^32^.

### Statistical analysis

The results are presented as the mean ± SD and are from three independent experiments. All data were analyzed by Prism (GraphPad Software, San Diego, CA, USA). Statistical differences between the two groups were determined using a two-tailed unpaired Student's *t*-test or a two-tailed nonparametric Mann–Whitney test. One-way analysis of variance (ANOVA) and Tukey’s post hoc test was used for comparing multiple groups. *P* values < 0.05 were considered statistically significant.

## Results

### Physical exercise attenuates the development of IVDD

**Fig. 1:**
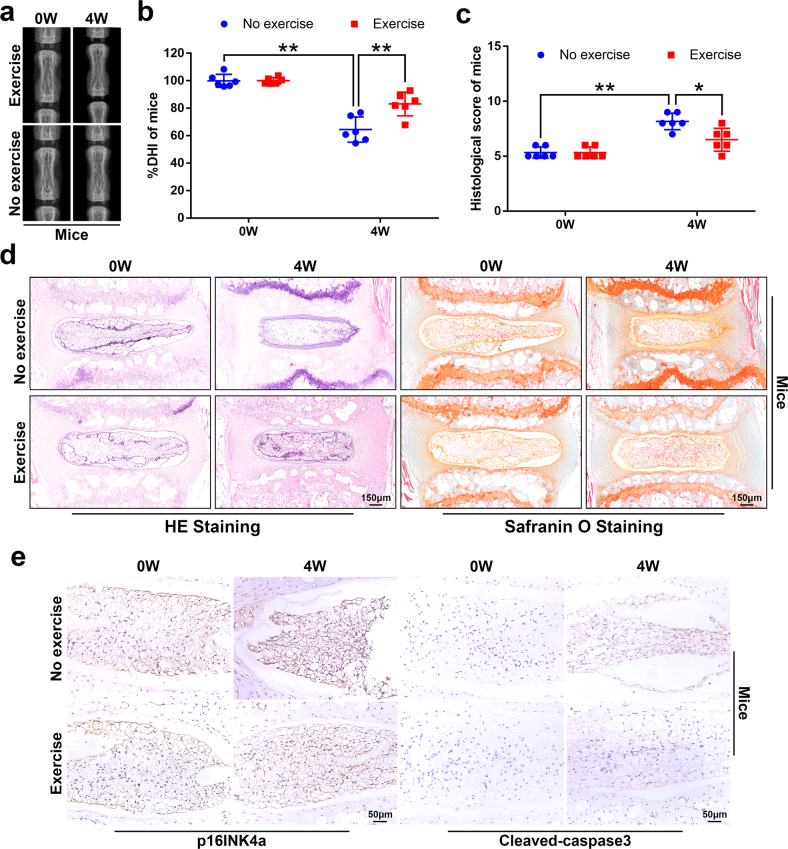
Physical exercise attenuates the development of IVDD. **a** Representative X-ray images of the mouse tail intervertebral space at 0 and 4 weeks. **b** The disc height index of a mouse tail disc at 0 and 4 weeks (*N* = 6). **c** The histological grades were evaluated at 0 and 4 weeks in mice. **d** Representative HE staining and SO staining of discs in mice from two experimental groups at 0 and 4 weeks (bar: 150 μm). **e** The respective immunohistochemical staining of p16INK4a and cleaved-caspase3 in NP tissues of mice (bar: 50 μm). All data are shown as the mean ± SD. *p < 0.05, **p < 0.01.

First, we sought to investigate whether physical exercise is capable of attenuating age-related IVDD in mice and rats. As shown by X-rays, after 4 weeks of physical exercise, the height of the intervertebral space of mice and rats in the exercise group was significantly higher than that of the no-exercise group (Fig. 1a, b and Supplementary Fig. 1a, b). Moreover, the results of HE staining (used to examine the general histological structure of IVD) showed that after 4 weeks of exercise, the number of cells in the NP and the structural integrity of the AF of the exercise group were better than those in the no-exercise group (Fig. 1c, d and Supplementary Fig. 1c, d). Additionally, SO staining (used to stain proteoglycans and glycosaminoglycans) showed that physical exercise had no significant effect on the extracellular matrix of the NP tissue (Fig. 1d and Supplementary Fig. 1d). On the other hand, apoptosis and senescence are closely related to IVDD^9,33^. To verify whether physical exercise could attenuate IVDD by alleviating the senescence and apoptosis of NP tissues, we detected the expression levels of p16INK4a and cleaved-caspase3 in NP tissues. The results of immunohistochemistry showed that after 4 weeks of physical exercise, the expression of p16INK4a and cleaved-caspase3 in the NP tissues of mice and rats in the exercise group was less than that in the no-exercise group (Fig. 1e and Supplementary Fig. 1e). In summary, these results indicate that physical exercise effectively attenuates the development of IVDD.

### Knockout of FNDC5 attenuates the effects of physical exercise on IVDD in vivo

Irisin is hydrolyzed from FNDC5, so immunological methods for detecting irisin inevitably detect its precursor FNDC5^29^. Moreover, due to dimerization and/or glycosylation^34,35^, the apparent molecular mass of irisin is 22–32 kDa, which is similar to the molecular mass of FNDC5^22,34^. Therefore, we refer to FNDC5/irisin when describing the immunoassay based on irisin. Since irisin is believed to constitute most of the secreted FNDC5/irisin^22^, we refer to irisin when describing the results obtained in NP or plasma using an irisin enzyme-linked immunosorbent assay (ELISA) kit.

**Fig. 2:**
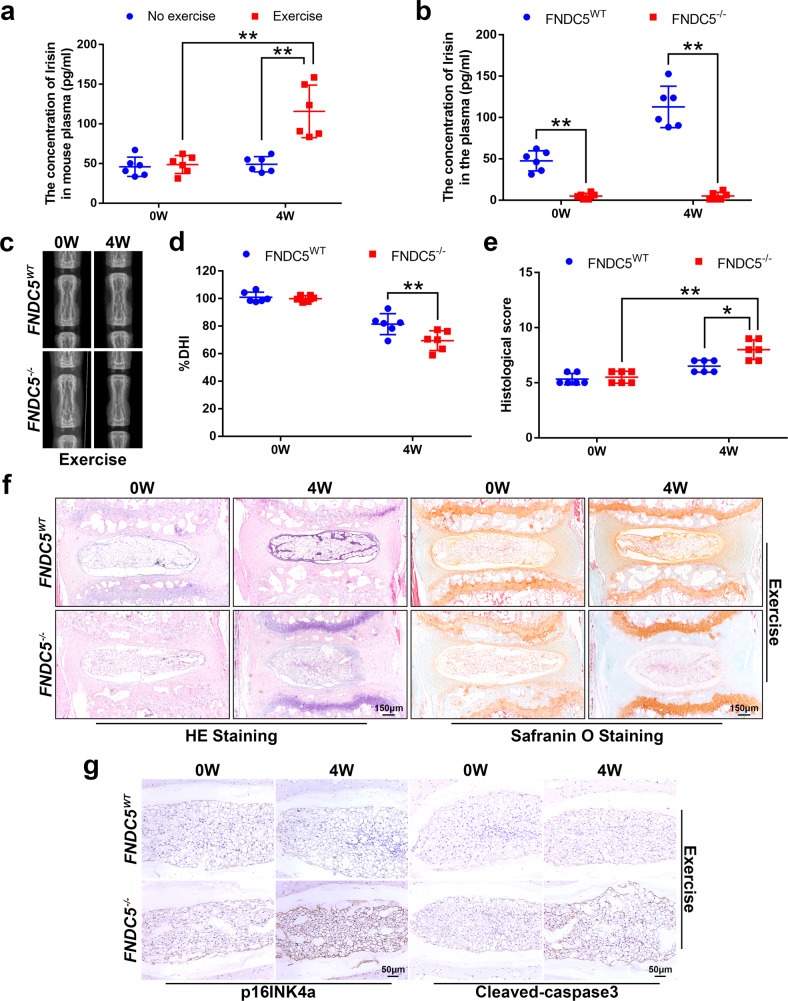
FNDC5/irisin plays a key role in the effect of physical exercise on IVDD. **a** Plasma content of irisin in the exercise group of mice compared to the no-exercise group (*N* = 6). **b** Plasma content of irisin in FNDC5^−/−^ mice compared to FNDC5^WT^ mice after 4 weeks of physical exercise (*N* = 6). **c** Representative X-ray images of the mouse tail intervertebral space at 0 and 4 weeks. **d** The disc height index of a mouse tail disc at 0 and 4 weeks (*N* = 6). **e** The respective histological grades of a mouse tail disc (*N* = 6). **f** Representative HE staining and SO staining of discs in FNDC5^−/−^ and FNDC5^WT^ mice (bar: 150 μm). **g** The respective immunohistochemical staining of p16INK4a and cleaved-caspase3 in NP tissues of FNDC5^WT^/FNDC5^−/−^ mice (bar: 50 μm). All data are shown as the mean ± SD. *p < 0.05, **p < 0.01.

As shown in Fig. 2a and Supplementary Fig. 2a, physical exercise effectively increased the content of irisin in the plasma of mice and rats. The content of irisin in the NP tissue of rats also increased after 4 weeks of physical exercise (Supplementary Fig. 2b). Because of limitations in the amount of mouse NP tissue, we were unable to determine the local content of irisin.

To determine whether irisin is a key factor for physical exercise to affect IVDD, we compared the changes in IVDs in FNDC5-knockout (FNDC5^−/−^) mice and wild-type (FNDC5^WT^) mice after 4 weeks of physical exercise. After knocking out FNDC5, we found that the plasma irisin content was significantly reduced, and even after 4 weeks of physical exercise, there was no change (Fig. 2b). In addition, comparing the changes in intervertebral space height in FNDC5^WT^ mice and FNDC5^−/−^ mice, we found that the benefit of physical exercise on the changes in intervertebral space height was compromised (Fig. 2c, d). The results of HE and SO staining showed that after 4 weeks of physical exercise, the structural integrity of NP and AF in FNDC5^WT^ mice was better than that of FNDC5^−/−^ mice, but the extracellular matrix of NP tissue did not change significantly (Fig. 2e, f). As shown in Fig. 2g, the beneficial effect of physical exercise on inhibiting the expression of p16INK4a and cleaved-caspase3 in NP tissue was compromised after knocking out FNDC5. Taken together, these data indicate that FNDC5/irisin is a key factor by which physical exercise attenuates the development of IVDD.

### FNDC5/irisin suppresses apoptosis and senescence in NP cells in vitro

**Fig. 3:**
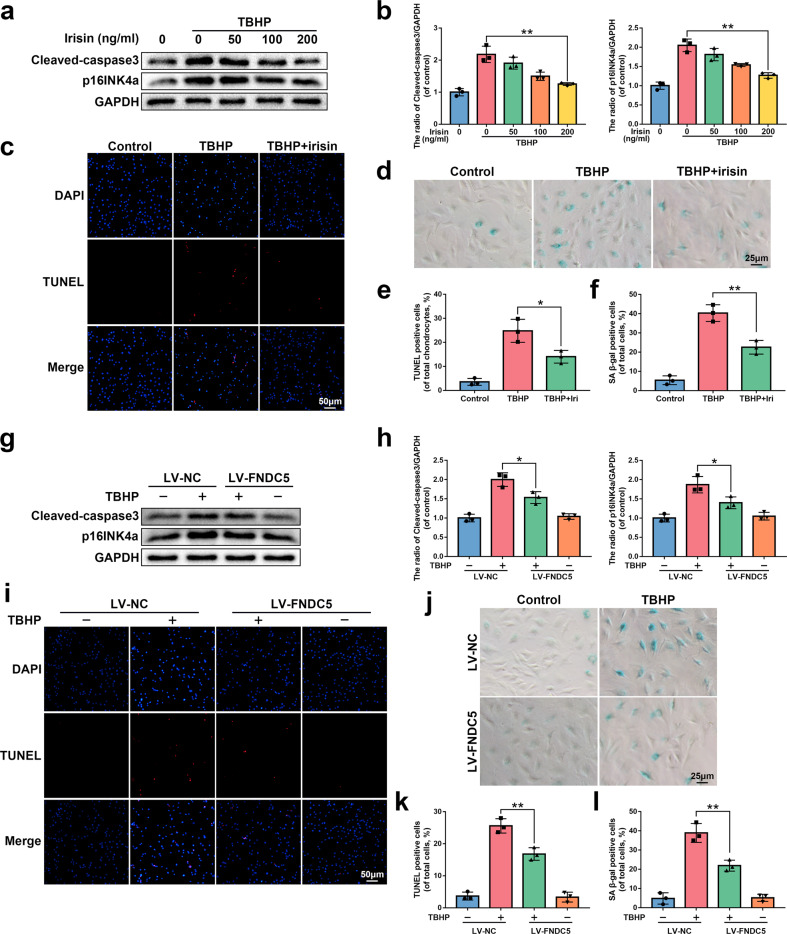
The protective effect of irisin and FNDC5 overexpression on NP cells in vitro. **a**, **b** The protein expression of p16INK4a and cleaved-caspase3 was detected by western blot in rat NP cells treated with or without 0, 50, 100, and 200 ng/ml irisin for 24 h and 60 μM TBHP for 24 h (*N* = 3). **c**, **e** The results of the TUNEL staining assay show the number of TUNEL-positive rat NP cells treated with or without 200 ng/ml irisin for 24 h and 60 μM TBHP for 24 h (bar: 50 μm, *N* = 3). **d**, **f** The results of the SA-β-gal staining assay show the number of SA-β-gal-positive rat NP cells treated as described above (bar: 25 μm, *N* = 3). **g**, **h** The protein expression of p16INK4a and cleaved-caspase3 was detected by western blot in rat NP cells transfected with LV-NC or LV-FNDC5 before receiving 60 μM TBHP (*N* = 3). **i**, **k** The results of the TUNEL staining assay show the number of TUNEL-positive rat NP cells treated as described above (bar: 50 μm, *N* = 3). **j**, **l** The results of the SA-β-gal staining assay show the number of SA-β-gal-positive rat NP cells treated as described above (bar: 25 μm, *N* = 3). All data are shown as the mean ± SD. *p < 0.05, **p < 0.01.

Oxidative stress exists in the entire pathophysiological process of IVDD^9,36^. We exposed NP cells to tert-butyl hydroperoxide (TBHP), an exogenous reactive oxygen species donor, to establish an in vitro IVDD model. The results of the cell counting kit-8 (CCK8) assay showed that TBHP inhibited the viability of NP cells (Supplementary Fig. 3a). We verified whether irisin (8880-IR-025, R&D systems) protects NP cells from senescence by SA-β-gal activity and p16INK4a expression, which are commonly used indicators of senescence. Cleaved-caspase3 expression and TUNEL staining (used to detect the level of DNA damage) were used to evaluate the anti-apoptotic effect of irisin. As shown in Fig. 3a–f, irisin inhibited the increased expression of p16INK4a and cleaved-caspase3 induced by TBHP and the increased SA-β-gal activity and DNA damage caused by TBHP.

Next, we transfected NP cells with lentivirus FNDC5 (LV-FNDC5) to upregulate the expression of FNDC5/irisin. The efficiency of lentiviral transfection was confirmed by western blot (Supplementary Fig. 3b). Similar to the previous results, overexpression of FNDC5 effectively prevented the increased expression of p16INK4a and cleaved-caspase3 and increased SA-β-gal activity and DNA damage caused by TBHP (Fig. 3g–l). Overall, these data suggest that irisin and FNDC5 overexpression protect NP cells from TBHP-induced senescence and apoptosis.

### FNDC5/irisin promotes autophagy in NP cells in vitro and in vivo

Autophagy is a protective mechanism that breaks down misfolded proteins and damaged organelles in the cell^37^. It participates in maintaining various physiological reactions and plays an important role in cell homeostasis^38^. On the other hand, research results suggested that physical exercise can induce autophagy in muscles, liver, and brain^39,40^. Therefore, we investigated whether FNDC5/irisin can alleviate senescence and apoptosis by enhancing autophagy in NP cells.

**Fig. 4:**
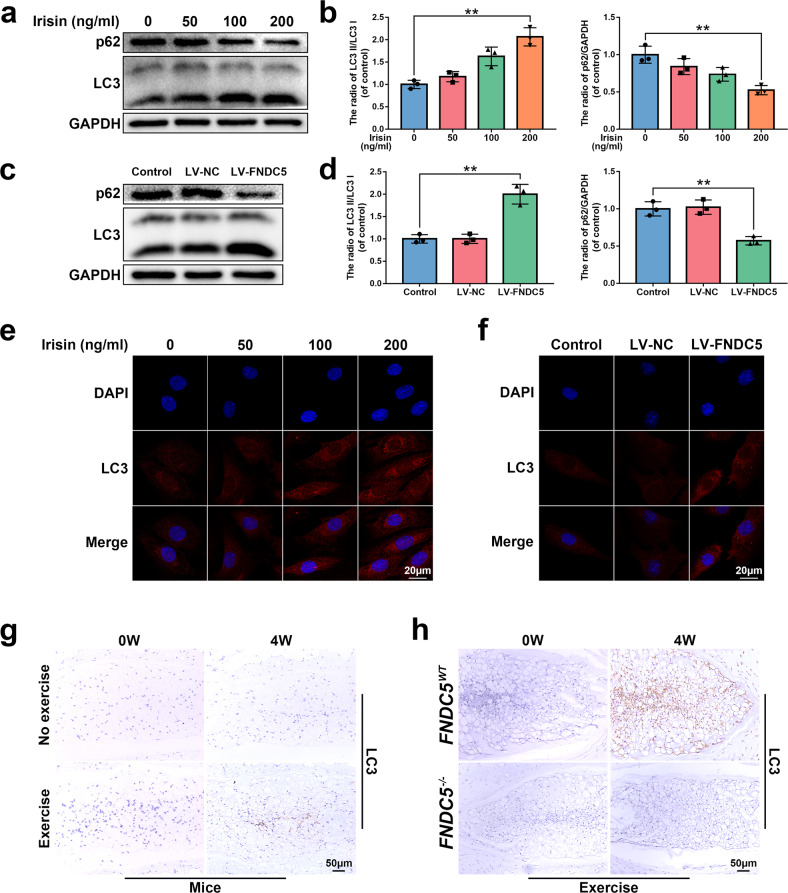
The effect of FNDC5/irisin on autophagy in vivo and in vitro. **a**, **b** The protein expression of p62 and LC3 was detected by western blot in rat NP cells treated with 0, 50, 100, and 200 ng/ml irisin for 24 h (*N* = 3). **c**, **d** The protein expression of p62 and LC3 was detected by western blot in rat NP cells transfected with LV-NC or LV-FNDC5 (*N* = 3). **e**, **f** Immunofluorescence staining of LC3 in rat NP cells treated as described above (bar: 20 μm). **g** The respective immunohistochemical staining of LC3 in NP tissues of mice (bar: 50 μm). **h** Immunohistochemical staining of LC3 in NP tissues from FNDC5^WT^/FNDC5^−/−^ mice (bar: 50 μm). All data are shown as the mean ± SD. *p < 0.05, **p < 0.01.

From the western blot results, as the concentration of irisin increased, the expression of p62 decreased, and the ratio of LC3II/I increased in NP cells, which are autophagy markers (Fig. 4a, b). The same result was found for LC3II (Fig. 4e). Additionally, the overexpression of FNDC5 in NP cells also showed the same result (Fig. 4c, d, f). Subsequently, we investigated the changes in LC3II in the NP tissues of mice and rats after physical exercise and found that physical exercise increased the expression of LC3II in the nucleus pulposus tissue (Fig. 4g and Supplementary Fig. 2c), but this increase was reversed after knocking out FNDC5 (Fig. 4h). Collectively, these data suggest that FNDC5/irisin promotes autophagy in NP cells in vitro and in NP tissues in vivo.

In addition, we investigated whether FNDC5/irisin activates autophagy through the AMPK/mTOR signaling pathway, which is a classic autophagy pathway^41^. As shown in Supplementary Fig. 4, irisin and FNDC5 overexpression could activate the AMPK/mTOR signaling pathway in NP cells; physical exercise could also increase the expression of p-AMPK in NP tissue, and after knocking out FNDC5, the expression of p-AMPK decreased.

### FNDC5/irisin suppresses apoptosis and senescence through autophagy

**Fig. 5:**
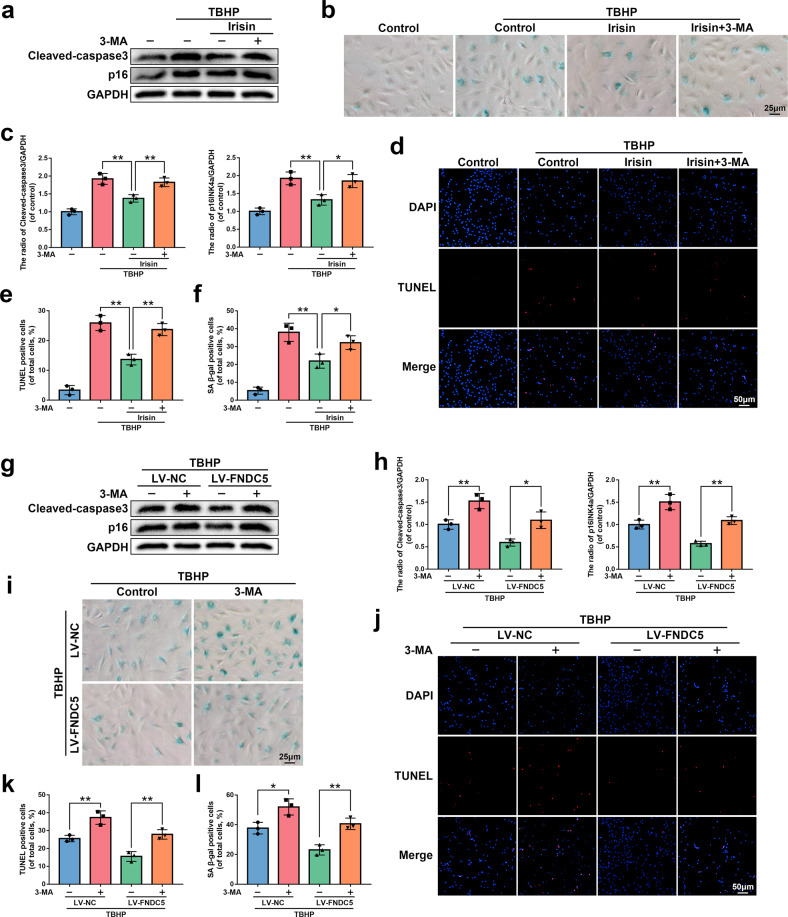
FNDC5/irisin suppresses apoptosis and senescence through autophagy. **a**, **c** The protein expression of p16INK4a and cleaved-caspase3 was detected by western blot in rat NP cells untreated, treated with TBHP (60 μM for 24 h) alone, treated with irisin (200 ng/ml for 24 h) and TBHP, or treated with 3-MA (10 mM for 2 h) before TBHP and irisin addition (*N* = 3). **b**, **f** The results of the SA-β-gal staining assay show the number of SA-β-gal-positive rat NP cells treated as described above (bar: 25 μm, *N* = 3). **d**, **e** The results of the TUNEL staining assay show the number of TUNEL-positive rat NP cells treated as described above (bar: 50 μm, *N* = 3). **g**, **h** The protein expression of p16INK4a and cleaved-caspase3 was detected by western blot in rat NP cells transfected with LV-NC or LV-FNDC5 and then pretreated with 10 mM for 2 h before receiving 60 μM TBHP (*N* = 3). **i**, **l** The results of the SA-β-gal staining assay show the number of SA-β-gal-positive rat NP cells treated as described above (bar: 25 μm, *N* = 3). **j**, **k** The results of the TUNEL staining assay show the number of TUNEL-positive rat NP cells treated as described above (bar: 50 μm, *N* = 3). All data are shown as the mean ± SD. *p < 0.05, **p < 0.01.

To further prove that FNDC5/irisin protects NP cells from senescence and apoptosis by activating autophagy, we used 3-methyladenine (3-MA), an inhibitor of the early stage of autophagy, to block autophagy. As shown in Fig. 5, the protein expression of p16INK4a and cleaved-caspase3 and the percentage of TUNEL- and SA-β-gal-positive cells all indicate that 3-MA could attenuate or eliminate the protective effect of FNDC5/irisin on TBHP-induced senescence and apoptosis. The above results demonstrate that FNDC5/irisin suppresses apoptosis and senescence through autophagy.

### FNDC5 overexpression ameliorates IVDD in rats in vivo

**Fig. 6:**
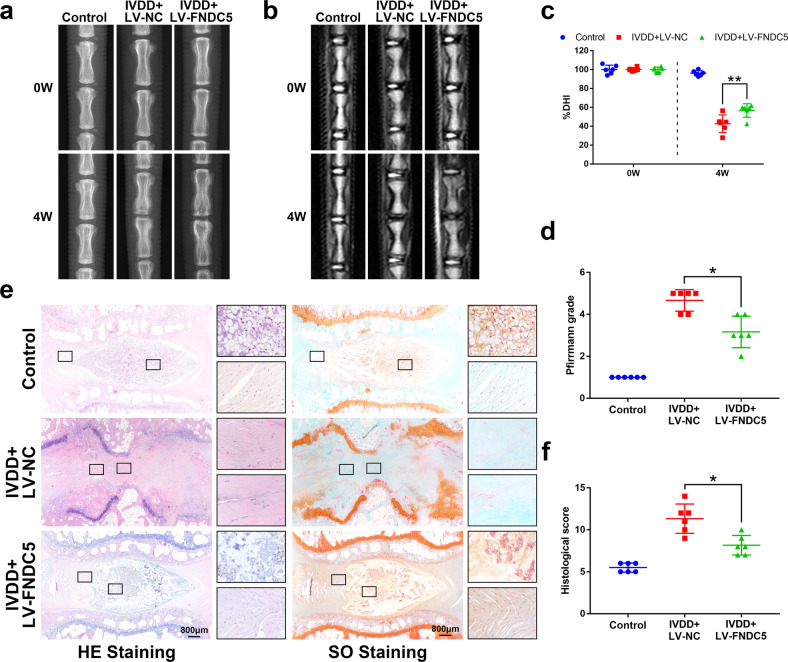
FNDC5/irisin ameliorates IVDD in rats in vivo. **a** Representative image of an X-ray of a rat tail disc at 0 and 4 weeks after disc puncture surgery. **b** Representative images of T2-weighted MRI of a rat-tail disc at 0 and 4 weeks after disc puncture surgery. **c** The disc height index of a rat-tail disc at 0 and 4 weeks after disc puncture surgery (*N* = 6). **d** Respective Pfirrmann grade scores at 0 and 4 weeks after disc puncture surgery (*N* = 6). **e** Representative HE staining and SO staining of discs from three experimental groups at 4 weeks (bar: 800 μm). **f** The histological grades evaluated at 4 weeks postsurgery in the three groups (*N* = 6). All data are shown as the mean ± SD. *p < 0.05, **p < 0.01.

To investigate the therapeutic effect of FNDC5/irisin on the rat IVDD model, lentivirus was injected into the rat IVD after IVDD surgery to overexpress FNDC5. X-ray, MRI, HE and SO staining was used to evaluate the imaging and histomorphological changes. The FNDC5 mRNA level in NP tissues in the IVDD + LV-FNDC5 group increased 14 days after lentivirus injection (Supplementary Fig. 5b). Through X-ray analysis, we found that after puncture surgery (IVDD surgery), the height of the intervertebral space was significantly lost, and FNDC5 overexpression significantly reversed this change (Fig. 6a, c). Moreover, magnetic resonance imaging (MRI) showed that 4 weeks after a puncture, the T2-weighted signal intensity of the injured IVD in the IVDD + LV-NC group disappeared, while the IVDD + LV-FNDC5 group showed a gray signal (Fig. 6b). The Pfirrmann grade scores (used to indicate the degree of IVDD) showed that the score of the IVDD + LV-FNDC5 group was significantly lower than that of the IVDD + LV-NC group (Fig. 6d).

From the results of HE and SO staining in Fig. 6e, it could be found that the NP tissue structure in the IVDD + LV-NC group disappeared, and the AF structure was disordered, while overexpression of FNDC5 could significantly improve the pathological changes of these tissues. The histological score also proved that overexpression of FNDC5 attenuates the development of IVDD (Fig. 6f). The above results demonstrate that FNDC5/irisin ameliorates IVDD in rats in vivo.

## Discussion

In most international guidelines for low back pain management, physical exercise is considered to be an effective treatment for patients with degenerative disc disease, which could effectively relieve pain and recover impaired motor function^42,43^. Previous studies mainly focused on the strengthening and stabilization of spine structures by exercise from the perspective of biomechanics^44^. However, there is little discussion on how physical exercise affects intervertebral disc degeneration at the cellular level and the underlying mechanism. Regular physical exercise is considered to be an effective autophagy inducer that can enhance autophagy activity in adipose tissue, the heart, the liver, and the brain^39,40,45^.

Irisin was originally discovered by Bostrom et al., as an exercise-induced myokine that promotes adipose metabolism^22,46^. An interesting study has shown that after humans undergo physical exercise, the concentration of irisin in the circulation could be increased^47^. Extending these previous findings and demonstrating the role of FNDC5/irisin in physical exercise affecting IVDD, we have confirmed for the first time from the perspective of myokine that physical exercise regulates the level of NP autophagy through FNDC5/irisin, thereby attenuating the development of IVDD.

In the animal model of physical exercise reported in this study, the improvement of intervertebral space height and IVD morphology, as well as the reduction of p16INK4a and cleaved-caspase3 (senescence and apoptosis-related proteins) expression, support the notion that physical exercise attenuates the development of IVDD^15,16^.

Irisin increased significantly in the circulation of mice and rats after physical exercise, suggesting that FNDC5/irisin may be an important factor for physical exercise to affect IVDD. Notably, compared with the spleen (a previous study showed that FNDC5 mRNA is highly expressed in muscle and expressed at low levels in the spleen^23^), FNDC5 mRNA was barely expressed in the NP (Supplementary Fig. 5a), indicating that exercise-induced irisin may be a key factor in the crosstalk between muscle and NP.

After knocking out FNDC5 in mice, the effects of physical exercise on the increase in circulating irisin, the improvement in the height of the intervertebral space, and the alleviation of the senescence and apoptosis of the NP tissue were compromised, indicating that physical exercise improves IVDD through the myokine irisin.

In in vivo and in vitro experiments, FNDC5/irisin effectively increased the autophagy level of the NP to support the notion that physical exercise regulates tissue autophagy through myokine^45,48^. Our finding that blockade of the autophagy of NP cells in vitro inhibited the anti-senescent and anti-apoptotic effects of FNDC5/irisin suggests that FNDC5/irisin attenuates the development of IVDD through autophagy. Unfortunately, we could not establish an in vivo autophagy blocking model to further verify this conclusion, nor could we establish a direct interaction between FNDC5/irisin and the AMPK/mTOR signaling pathway to prove that FNDC5/irisin activates autophagy through the AMPK/mTOR signaling pathway.

In two interesting studies, Storlino et al. and He et al. found that moderate irisin levels can promote bone anabolism and inhibit the apoptosis of osteocytes^26,27^. Vertebral subchondral bone is part of the vertebral endplate and maintains the integrity of the intervertebral disc by preventing the highly hydrated NP from penetrating the adjacent vertebral body and maintaining the nutrition of the endplate and the intervertebral disc^28,49–51^. We evaluated the apoptosis of VSB by detecting the expression level of cleaved-caspase3 in VSB. As shown in Supplementary Fig. 6, after 4 weeks of exercise, the expression of cleaved caspase-3 in the VSB was reduced, but this effect was attenuated after the knockdown of FNDC5. This finding provides a new research idea for FNDC5/irisin to delay IVDD; that is, FNDC5/irisin attenuates IVDD by affecting vertebral subchondral bone (VSB). Subsequent studies can improve the function of FNDC5/irisin in attenuating IVDD.

Our findings suggest that FNDC5/irisin could comprise an attractive novel therapy aimed at delaying the progression of IVDD, including for those who can no longer exercise. Many patients with IVDD refuse to perform regular physical exercise because they often have LBP or other age-related diseases (such as heart disease, osteoarthritis, and obesity). Therefore, the development of alternative methods based on the beneficial effects of physical exercise on IVDs may benefit these patients, such as the use of drugs to specifically increase FNDC5/irisin.

In this study, FNDC5/irisin was overexpressed in NP by intervertebral injection of lentivirus, demonstrating that FNDC5/irisin has a protective effect on the intervertebral disc. Since irisin is currently not an FDA-approved drug, follow-up studies can focus on screening the FDA drug library for drugs that specifically increase muscle secretion of irisin.

In conclusion, our results show for the first time from the perspective of myokines that physical exercise activates autophagy by increasing FNDC5/irisin, thereby delaying the progression of IVDD. We further proved that FNDC5/irisin is a novel mediator of the beneficial effects of physical exercise on senescence and apoptosis in IVDD models. Increasing the level of FNDC5/irisin, either pharmacologically or through physical exercise, maybe a novel therapeutic strategy to protect the NP from senescence and apoptosis and prevent LBP in IVDD.

## Supplementary information

Supplemental information
