# Supplementary material for: Exercise-induced FNDC5/irisin protects nucleus pulposus cells against senescence and apoptosis by activating autophagy
Source: Exp Mol Med. 2022 Jul 26;54(7):1038–48. doi: 10.1038/s12276-022-00811-2 (PMC9355987; doi:10.1038/s12276-022-00811-2)
Supplement: Supplementary file 1 — Supplemental information [file 12276_2022_811_MOESM1_ESM.pdf]

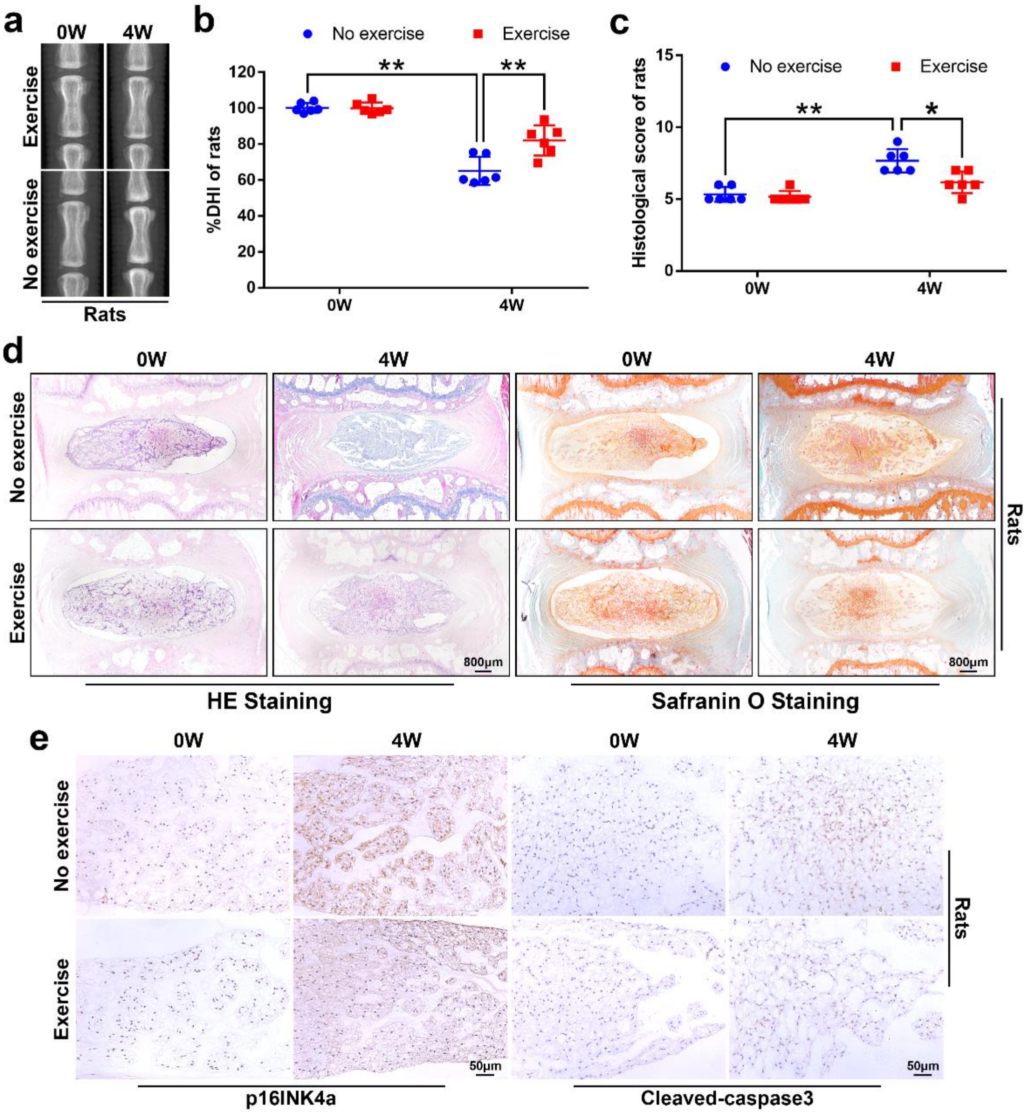

Supplementary Fig.1 a. Representative images of X-ray of rat-tail intervertebral space at 0 and 4 weeks. **b.** The disc height index of a mice-tail disc at 0 and 4 weeks (N=6). **c.** The histological grades evaluated at 0 and 4 weeks in rats. **d.** Representative HE staining and SO staining of discs in rats from two experimental groups at 0 and 4 weeks (bar: 800μm). **e.** The respective immunohistochemical staining of p16INK4a and cleaved-caspase3 in NP tissues of rats (bar:50μm). All data were shown as mean ± SD. *p < 0.05, **p < 0.01.

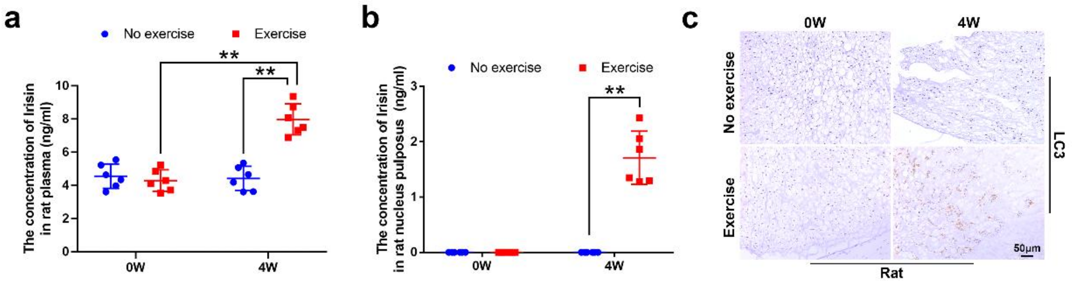

Supplementary Fig.2 a. Plasma content of irisin in the exercise group of rats compared to no-exercise group (N=6). **b.** NP tissue content of irisin in the exercise group of rats compared to no-exercise group (N=6). **c.** The respective immunohistochemical staining of LC3 in NP tissues of rats (bar:50μm). All data were shown as mean ± SD. *p < 0.05, **p < 0.01.

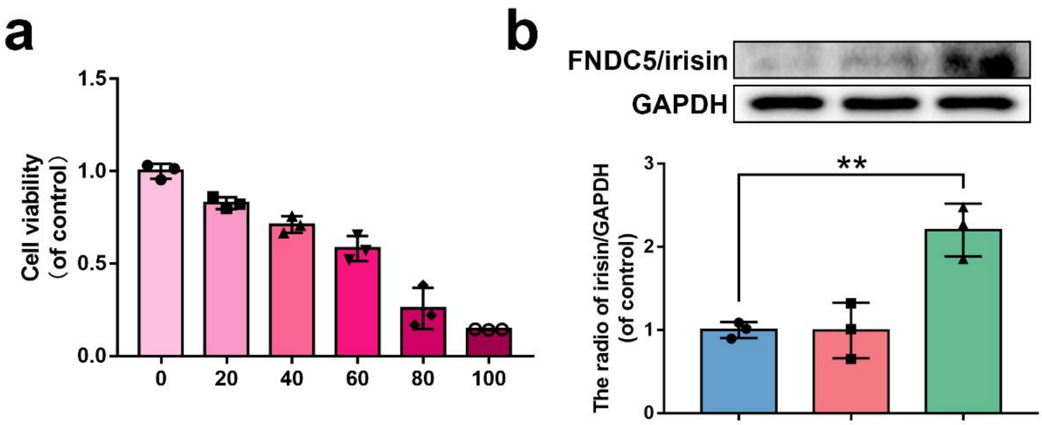

Supplementary Fig.3 a. The results of CCK-8 assay show the viability of NP cells treated with different concentrations of TBHP for 24h (N=3). **b.** The protein expression of FNDC5/irisin was detected by western blot in rat NP cells by transfecting with lentivirus (N=3). All data were shown as mean ± SD. *p < 0.05, **p < 0.01.

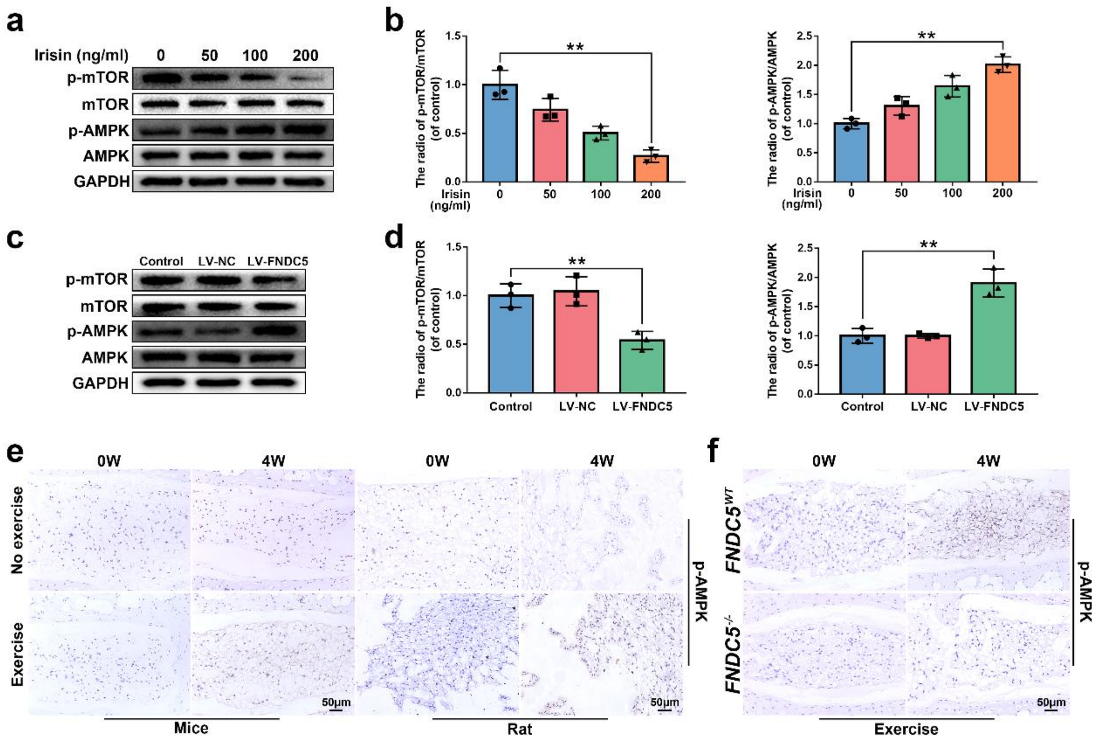

Supplementary Fig.4 a, b. The protein expression of p-mTOR, mTOR, p-AMPK and AMPK was detected by western blot in rat NP cells treated with 0, 50, 100, and 200ng/ml irisin for 24h (N=3). **c, d.** The protein expression of p-mTOR, mTOR, p-AMPK and AMPK was detected by western blot in rat NP cells transfected with LV-NC or LV-FNDC5 (N=3). **e.** The respective immunohistochemical staining of p-AMPK in NP tissues of mice and rats (bar:50μm). **f**. The respective immunohistochemical staining of p-AMPK in NP tissues of FNDC5^{WT} /FNDC5$^{-/-}$ mice (bar:50μm). All data were shown as mean ± SD. *p < 0.05, **p < 0.01.

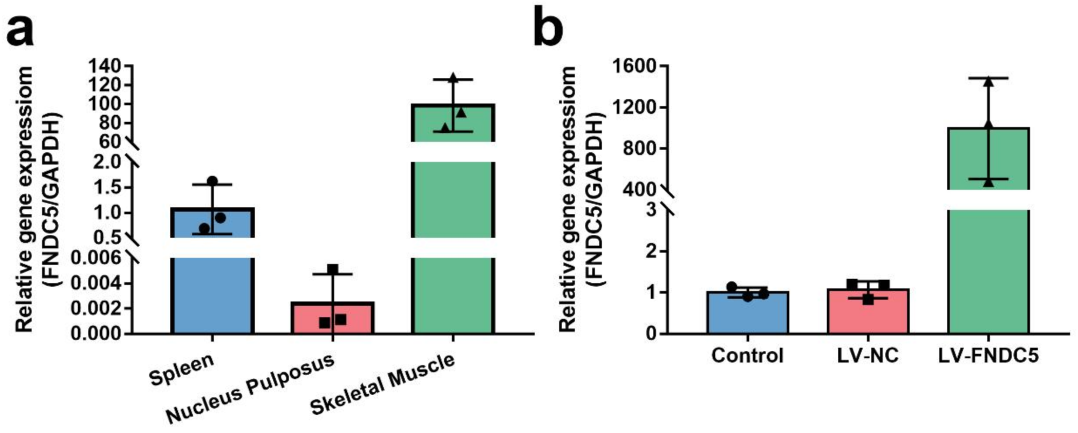

Supplementary Fig.5 a. The gene expression of FNDC5 in rat spleen, NP, and skeletal muscle

(N=3). **b.** The gene expression of FNDC5 in rat NP tissue at 14days after lentivirus injection (N=3). All data were shown as mean ± SD. *p < 0.05, **p < 0.01.

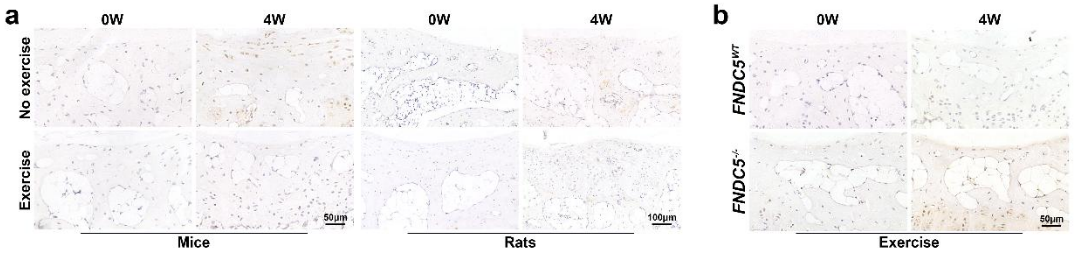

Supplementary Fig.6 a. The respective immunohistochemical staining of cleaved-caspase3 in VSB tissues of mice and rats (the bar of mice: 50μm; the bar of rats: 100μm). **b.** The respective immunohistochemical staining of cleaved-caspase3 in VSB tissues of $FNDC5^{WT}$ /$FNDC5^{-/-}$ mice (bar:50μm).

Supplementary Table 1

Primers used for mRNA expression studies using PCR

Gene	Forward primer (5'->3')	Reverse primer (5'->3')	Origin
FNDC5	GATCATCGTCGTGGTCCTCTT	TGTTATTGGGCTCGTTGTCCT	Rat
GAPDH	GGCTCTCTGCTCCTCCC	CCGTTCACACCGACCTT	Rat

cyagen

We help you discover life

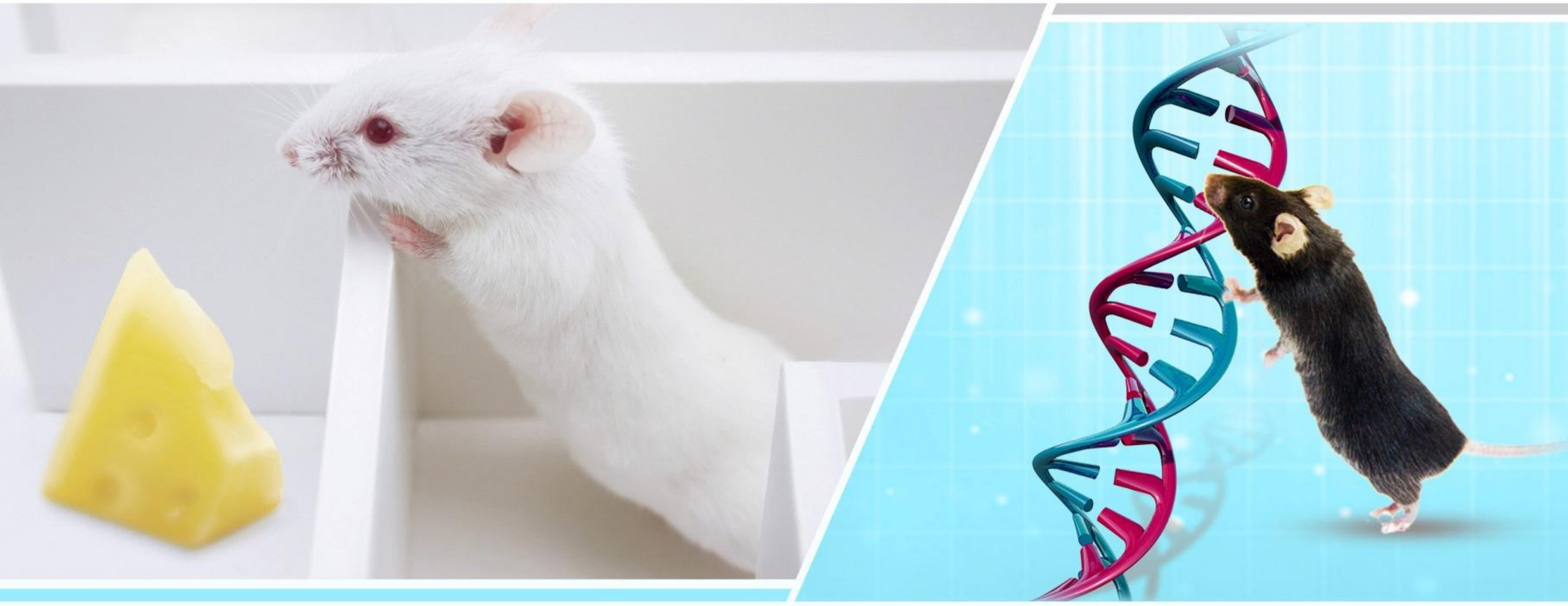

Genotyping Strategy

Contract No: KOAIP200429YZ1-B

- Confidential -

Cyagen Biosciences Inc.

www.cyagen.com

www.cyagen.com
Tel: 800-921-8930; Fax: 408-969-0336
2255 Martin Ave., Suite E. Santa Clara, CA 95050

1. Product Information

Name	C57BL/6N-Fndc5^{em1cyagen}
Serial Number	KOCMP-384061-Fndc5-B6N
Gene	Fndc5
NCBI ID	384061
Strain	C57BL/6N
Type	conventional knockout

2. Targeting Strategy

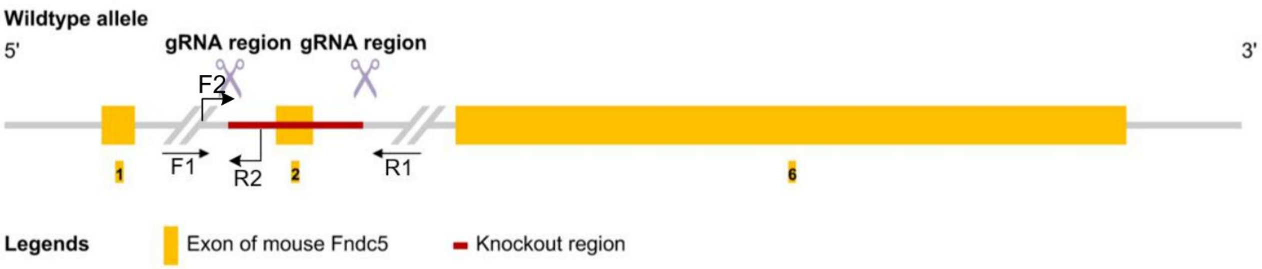

3. Genotyping strategy

Primers1: (Annealing Temperature 60.0 ºC)

F1: 5'-CTGTCTCCAATGTTCCACTTGTCTG-3'

R1: 5'-CTTGCCTTTGTTCTTTGAGGCCATC-3'

Product size: 815 bp   Wildtype allele: 1798 bp

Primers2: (Annealing Temperature 60.0 ºC)

F2: 5'-CACTTGTCTGCTCTCTGGTTCTGT-3'

R2: 5'-GCTTGAACCAAGGCGAGAGCTAGT-3'

Product size: 678 bp

Homozygous: one band with 815 bp
Heterozygous: three bands with 815 bp, 678 bp and 1798 bp
Wildtype allele: two bands with 678 bp and 1798 bp

Note: If DNA sample is not very pure or without enough PCR extension time, the 1798 bp PCR product may not be amplified.

4. Expected Results

Genotyping	Primers1	Primers2
Fndc5 (-/-): Homozygous	M WT Water<br>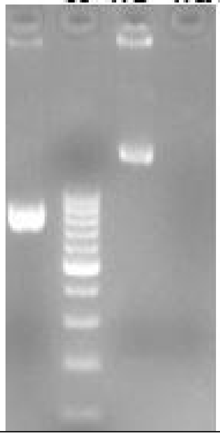	M WT Water<br>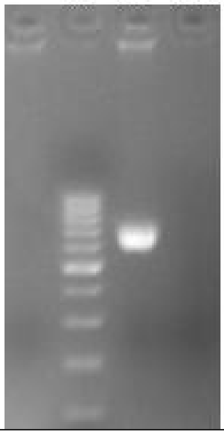

Note:

1) PCR was carried out in 25 μL volume for 35 cycles under standard conditions, with all two primers listed above added to each reaction.

2) DNA marker: Thermo Scientific GeneRuler 100 bp DNA Ladder #SM0242

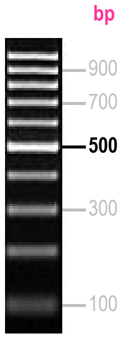

3) Controls used in PCR genotyping are:

- Water control: No DNA template added.
- Wildtype control: Mouse genomic DNA.

5. PCR reaction

5.1 DNA Extraction

- Method One:

We recommend that using TaKaRa MiniBEST Universal Genomic DNA Extraction kit (Ver.5.0_Code No. 9765) to gain high purity of genomic DNA.

a. Add 180 μL of Buffer GL, 20 μL of Proteinase K and 10 μL of RNase A per tail piece (2-5 mm) in a microcentrifuge tube. Be careful not to cut too much tail.

b. Incubate the tube at 56℃ overnight.

c. Spin in microcentrifuge at 12,000 rpm for 2 minutes to remove impurities.

d. Add 200 μL Buffer GB and 200 μL absolute ethyl alcohol with sufficient mixing.

e. Place the spin Column in a collection tube. Apply the sample to the spin and centrifuge at 12,000 rpm for 2 min. Discard flow-through.

f. Add 500 μL Buffer WA to the spin column and centrifuge at 12,000 rpm for 1 min. Discard flow-through.

g. Add 700 μL Buffer WB to the spin column and centrifuge at 12,000 rpm for 1 min. Discard flow-through. (Note: Make sure the Buffer WB has been premixed with 100% ethanol. When adding Buffer WB, add to the tube wall to wash off the residual salt.)

h. Repeat step g.

i. Place the spin Column in a collection tube and centrifuge at 12,000 rpm for 2 min.

j. Place the spin Column in a new 1.5ml tube. Add 50~200 μL sterilized water or elution buffer to the center of the column membrane and let the column stand 5min. (Note: Heating sterilized water or elution buffer up to 65℃ can increase the yield of elution.)

k. To elute DNA, centrifuge the column at 12,000 rpm for 2 min. To increase the yield of DNA, add the flow-through and/or 50~200 μL sterilized water or elution buffer to the center of the spin column membrane and let the column stand 5 min. Centrifuge at 12,000 rpm for 2 min.

l. Quantify to genomic DNA. Eluted genomic DNA can be quantified by electrophoresis or electrophoresis.

- Method Two:

A low-cost and sample method to gain rough genomic DNA.

a. Add 100 μL of tail digestion buffer per tail piece (2-5 mm) in a microcentrifuge tube. Be careful not to cut too much tail.

b. Incubate the tube at 56℃ overnight.

c. Incubate the tube at 98℃ for 13 minutes to denature the Proteinase K.

d. Spin in microcentrifuge at top speed for 15 minutes. Use an aliquot of supernatant straight from the tube (1 μL in a 12.5 μL reaction) for PCR.

Final concentration of tail digestion buffer:

- 50 mM KCl
- 10 mM Tris-HCl (pH 9.0)
- 0.1 % Triton X-100
- 0.4 mg/mL Proteinase K

5.2 PCR Mixture (primer concentration: 10μM):

Component	x1
ddH_2O	9.0 μl
Product primer F	1.0 μl
Product primer R	1.0 μl
Premix Taq	12.5 μl
DNA	1.5 μl
Total	25 μl

5.3 PCR Reaction Conditions:

Step	Temp.	Time	Cycles
Initial denaturation	94 °C	3 min	
Denaturation	94 °C	30 s	35 x
Annealing	60 °C	35 s	
Extension	72 °C	35 s	
Additional extension	72 °C	5 min	

5.4 Relevant Reagents:

**Trizma Hydrochloride Solution**	Sigma, Cat. No. T2663
**Proteinase K**	Merck, Cat. No. MK539480
**Triton X-100**	Sigma, T8787-50 mL
**2 × Taq Master Mix (Dye Plus)**	Vazyme, P222
**Agarose**	BIOWEST AGAROSE, REGULAR
**DNA Marker**	Thermo Scientific GeneRuler 100 bp DNA Ladder #SM0242
**0.5×TBE**	Tris Bio Basic Inc, TBO194-500g
	EDTA Shanghai Sangon, 0105-500g
	Boric Acid, Shanghai Sangon, 0588-500g
